# Sympathetic nervous dysregulation in the absence of systolic left ventricular dysfunction in a rat model of insulin resistance with hyperglycemia

**DOI:** 10.1186/1475-2840-10-75

**Published:** 2011-08-10

**Authors:** James T Thackeray, Jerry Radziuk, Mary-Ellen Harper, Erik J Suuronen, Kathryn J Ascah, Rob S Beanlands, Jean N DaSilva

**Affiliations:** 1 Molecular Function & Imaging Program, National Cardiac PET Centre, University of Ottawa Heart Institute, 40 Ruskin Street, Ottawa, K1Y 4W7, Canada; 2 Division of Cardiology, University of Ottawa Heart Institute, 40 Ruskin Street, Ottawa, K1Y 4W7, Canada; 3 Ottawa Hospital Research Institute, 725 Parkdale Avenue, Ottawa, K1Y 4E9, Canada; 4 Department of Cellular & Molecular Medicine, Faculty of Graduate & Postdoctoral Studies, University of Ottawa, 451 Smyth Road, Ottawa, K1H 8M5, Canada; 5 Department of Biochemistry, Microbiology, and Immunology, Faculty of Graduate & Postdoctoral Studies, University of Ottawa, 451 Smyth Road, Ottawa, K1H 8M5, Canada

**Keywords:** norepinephrine, [^11^C]meta-hydroxyephedrine (HED), small animal echocardiography, positron emission tomography, diabetes mellitus, cardiovascular disease

## Abstract

**Background:**

Diabetes mellitus is strongly associated with cardiovascular dysfunction, derived in part from impairment of sympathetic nervous system signaling. Glucose, insulin, and non-esterified fatty acids are potent stimulants of sympathetic activity and norepinephrine (NE) release. We hypothesized that sustained hyperglycemia in the high fat diet-fed streptozotocin (STZ) rat model of sustained hyperglycemia with insulin resistance would exhibit progressive sympathetic nervous dysfunction in parallel with deteriorating myocardial systolic and/or diastolic function.

**Methods:**

Cardiac sympathetic nervous integrity was investigated *in vivo *via biodistribution of the positron emission tomography radiotracer and NE analogue [^11^C]*meta-*hydroxyephedrine ([^11^C]HED). Cardiac systolic and diastolic function was evaluated by echocardiography. Plasma and cardiac NE levels and NE reuptake transporter (NET) expression were evaluated as correlative measurements.

**Results:**

The animal model displays insulin resistance, sustained hyperglycemia, and progressive hypoinsulinemia. After 8 weeks of persistent hyperglycemia, there was a significant 13-25% reduction in [^11^C]HED retention in myocardium of STZ-treated hyperglycemic but not euglycemic rats as compared to controls. There was a parallel 17% reduction in immunoblot density for NE reuptake transporter, a 1.2 fold and 2.5 fold elevation of cardiac and plasma NE respectively, and no change in sympathetic nerve density. No change in ejection fraction or fractional area change was detected by echocardiography. Reduced heart rate, prolonged mitral valve deceleration time, and elevated transmitral early to atrial flow velocity ratio measured by pulse-wave Doppler in hyperglycemic rats suggest diastolic impairment of the left ventricle.

**Conclusions:**

Taken together, these data suggest that sustained hyperglycemia is associated with elevated myocardial NE content and dysregulation of sympathetic nervous system signaling in the absence of systolic impairment.

## Background

Diabetes mellitus, hyperglycemia, and insulin resistance confer a significantly greater risk of developing cardiovascular disease and heart failure [1]. The mechanisms involved in the progression of ventricular dysfunction in diabetes are diverse and unclear, with several pathways having been implicated including disrupted metabolic processes [2,3], glycation and interstitial fibrosis of myocardium [4], oxidative stress and apoptosis [5], and impairment of autonomic, particularly sympathetic, signal transduction [5-8].

The sympathetic nervous system is the primary extrinsic control of heart rate and contractility. Under stress, release of the neurotransmitter norepinephrine (NE) from sympathetic nerves enhances cardiac output [9]. Signaling is terminated by recovery of NE from the synaptic cleft back into the neuron varicosity via the NE reuptake transporter (NET) [10]. Dysregulation of sympathetic innervation has been documented in congestive heart failure [11], acute myocardial infarction [12], and diabetes [13]. Enhanced sympathetic signaling partially compensates for deteriorating myocardial function [9].

Glucose, insulin, and non-esterified fatty acids (NEFA) are potent stimulants of NE and epinephrine release [14,15]. Diabetic animals display reduced heart rate variability, hypertension, and elevated plasma and tissue levels of NE [16-20]. These animals develop systolic and diastolic cardiac dysfunction over time [4,18,19,21]. The frequent concurrence of diabetes with cardiac disease, with each increasing the risk of developing the other, implies a common pathogenesis [1]. It has been suggested that sympathetic dysregulation may be involved in such a process [1,7].

[^11^C]*meta*-Hydroxyephedrine ([^11^C]HED) has been routinely applied in cardiac positron emission tomography (PET) to interrogate sympathetic nervous integrity in a myriad of clinical conditions including acute myocardial infarction, cardiomyopathies, coronary artery disease, and heart failure [10,11]. Retention of [^11^C]HED is blocked by NET inhibitors and reduced by treatments elevating synaptic NE [22,23]. Altered uptake of [^11^C]HED and other presynaptic tracers has been described extensively in diabetic rats and mice [8,13], as well as clinically [7]. The temporal progression of cardiac sympathetic dysregulation and its relation to left ventricular dysfunction remains unclear.

We hypothesized that sustained hyperglycemia would contribute to cardiac sympathetic nervous dysfunction which would manifest in parallel with deteriorating cardiac systolic and diastolic function. The high fat diet-fed, moderate dose streptozotocin (STZ) rat model of sustained hyperglycemia with insulin resistance was serially characterized for metabolic and cardiac function, and sympathetic nervous integrity was tested at 2 and 8 weeks of unregulated hyperglycemia using *ex vivo *biodistribution of [^11^C]HED with NET expression and NE levels measured concurrently. This combined approach establishes an association between altered sympathetic nervous signaling and functional abnormalities during the progression of diabetes.

## Methods

### Drugs, chemicals, and radiochemistry

Desipramine hydrochloride, NE bitartrate, and STZ were obtained from Sigma-Aldrich (Toronto, ON, Canada). Drugs were dissolved in saline except STZ, which was in tribasic citrate. Antibodies against rat NET (AB5066P), tyrosine hydroxylase (AB1542) and GAPDH (sc32233) were obtained from Chemicon/Millipore (Billerca, MA, USA) and Santa Cruz Biotechnology (Santa Cruz, CA, USA), respectively. Secondary horseradish peroxidase conjugated IgG antibodies to mouse, rabbit and sheep were from Santa Cruz (sc2314, sc32004) or Abcam (ab6747, Cambridge, MA, USA). Histological stains were from Richard Allan Scientific (Kalamazoo, MI, USA). [^11^C]HED was synthesized as previously described and dissolved in 50:44:6 0.9% saline/water/8.4% sodium bicarbonate (v/v/v) for injection [23]. High radiochemical purity (> 99%) and specific activity (7.3-55.5 GBq/μmol) were obtained.

### Animal model

Animal experiments were conducted in accordance with the Canadian Council on Animal Care, with approval of the Animal Care Committee of the University of Ottawa. Adult male Sprague-Dawley rats (200-250 g) were obtained from Charles River Canada (Montreal, QC, Canada) and housed in a temperature-controlled animal facility under a 12 hour light/dark cycle with food and water *ad libitum*. Rats were maintained on high fat diet (Research Diets D12266B, New Brunswick, NJ, USA) composed of (by kJ) 32% fat, 51% carbohydrate, and 17% protein. After two weeks of initial feeding, a single intraperitoneal injection of moderate dose STZ (45 mg/kg, n = 99) or vehicle (n = 44) was administered [19,24-26]. Fed state blood glucose levels 2 weeks post-STZ were used to stratify treated rats by glycemic state, with blood glucose levels exceeding 11 mM considered to be hyperglycemic and the remainder considered as a STZ-treated euglycemic control group [19,24]. Body weights, diet consumption, and blood markers were assessed in all animals. Whole heart weights were determined at the conclusion of experiments and expressed as a ratio to body weight. STZ- and vehicle-treated rats were divided among several experimental protocols (Table 1). An additional group (n = 5) were fed standard rodent chow (Teklad 2019, Harlan Teklad, Madison, WI, USA) for euglycemic clamp experiments.

**Table 1:** Sample sizes of rat groups for individual experiments

		Streptozotocin	
	Controls	Euglycemic	Hyperglycemic
2 Weeks			
HED*	6	4	4
HED + Desipramine*	4	3	4
Western Blot*^†^	3	3	3
None*^†^	3	5	6

2 Weeks Total	16	16	16

8 Weeks			
HED*	8	9	14
HED + Desipramine*	6	6	5
OGTT + Western Blot*^†^	3	3	3
Echocardiography*^†^	5	7	8
Clamp + Histology*	3	5	4
Indirect Calorimetry*	3	0	3

8 Weeks Total	28	30	37

*Blood samples taken for measurement of biochemistry markers.

^†^Heart samples used for measurement of tissue norepinephrine.

### Blood markers

Fed state blood glucose was tested weekly from the saphenous vein using a glucose meter (AccuChek, Roche Diagnostics, Laval, QC, Canada). Fasted (overnight 8-12 h) plasma insulin, NEFA and triglyceride were measured from trunk blood collected at 2 and 8 weeks post-STZ following decapitation by radioimmune assay (Linco/Millipore, Billerca, MA, USA) or microplate colorimetric assays (Biovision, Mountain View, CA, USA), respectively.

### Plasma and cardiac norepinephrine

Plasma and reconstituted heart samples were analyzed for NE content using a modified inline capture, column-switching high performance liquid chromatography procedure with electrochemical detection [23,27]. Briefly, venous blood samples were centrifuged at 3000 × *g *to separate plasma and filtered (0.2 μm) for injection. Excised hearts were homogenized under 80/20 ethanol/0.1 M formic acid, centrifuged at 82000 × *g*, and supernatant evaporated in a rotary evaporator. The residue containing NE was reconstituted under 0.1 M formic acid and filtered. Samples were injected and NE adsorbed onto a capture column (Direct Connect refillable guard column, 2 × 20 mm, Alltech, Deerfield, IL, USA) containing activated aluminum oxide (Type WA4, Sigma) under 1.5 M Tris, 0.05 M EDTA (2 mL), rinsed with 10 mL of deionized water (1 mL/min), then eluted by 5/95 methanol/50 mM ammonium formate, 0.27 mM EDTA, 0.346 mM octanesulfonic acid (pH 2.85, 1 mL/min) onto a cation exchange analytical column (Partisil SCX, 250 × 4.6 mm, 10 μm, Phenomenex, Torrance, CA, USA) for separation (NE retention time = 6.8 min), and quantified by flow-through coulometric electrochemical detection (Coulochem III, ESA/Dionex, Chelmsford, MA, USA) with an applied voltage of 400 mV. Amperage signals were integrated using the PeakSimple Six Port Chromatography Data System and analysed where area under the curve is representative of mass NE. This procedure was validated using NE bitartrate standards, demonstrating 95% recovery and linear reproducibility in a physiological range (0.001-20 ng).

### Oral glucose tolerance

Serial oral glucose tolerance tests were performed as described elsewhere [25], at three intervals: prior to high fat feeding, 2 weeks after STZ or vehicle injection, and 8 weeks after STZ or vehicle injection.

### Euglycemic clamp

The hyperinsulinemic euglycemic clamp was performed as described elsewhere [3]. Continuous iv infusion of biosynthetic insulin (Novolin, 30 mU kg^-1 ^min^-1^) was counteracted by variable iv infusion of 20% glucose to maintain glycemia at 5 mM. Rats were considered clamped with three consecutive stable readings of blood glucose levels and glucose infusion rate, within 120 min.

### Indirect calorimetry

Calorimetric measures were obtained at 2 and 8 weeks of diabetes using the 4-chamber Oxymax system (Columbus Instruments, Columbus, OH, USA) as previously described [28]. Rats were sequestered in individual 11.7 L calorimetry chambers with food and water *ad libitum *for 24 h. Oxygen consumption, carbon dioxide production and respiratory exchange ratio (RER) were measured five times per hour. RER provides an index of carbohydrate versus fatty acid substrate utilization [28].

### Echocardiography

Serial echocardiography was carried out biweekly under light anesthesia (1-2% isoflurane) using the Vevo 770 system (VisualSonics, Toronto, ON, Canada) and a 23.5 MHz probe [3]. All echocardiography studies were performed and analyzed by a single operator. Parasternal long and short axis views were recorded as sequential ECG-gated M-mode sweeps (EKV-mode) to generate two-dimensional cines of the left ventricle. Endocardial and epicardial areas were traced on the two-dimensional parasternal long axis cine and used to calculate left ventricular volumes at end systole and end diastole. Calculations for stroke volume, percent ejection fraction (%EF), cardiac output, and percent fractional area change (%FAC) were completed using VisualSonics software. Diastolic function was assessed using pulse-wave Doppler across the mitral valve from the apical four chamber view [3]. Early (E) and late (A) flow velocity as well as mitral valve deceleration time provide an indication of diastolic function.

### [^11^C]HED ex vivo biodistribution

Biodistribution studies were performed at 2 and 8 weeks following STZ or vehicle injection in conscious animals, as described previously [23,28]. Briefly, 50-75 MBq (0.2-1.3 μg cold mass) of [^11^C]HED was injected as a 0.1-0.3 mL bolus into a lateral tail vein of restrained rats. Animals were killed by decapitation 30 min after tracer injection. A sample of trunk blood was collected. Hearts were rapidly excised and dissected to right and left atria, right and left ventricle free walls, and intraventricular septum, and a portion of skeletal muscle (quadriceps femoris) was taken as a reference tissue devoid of specific retention [23]. Samples were counted for radioactivity (decay corrected) in a gamma-counter along with 1% standard dilutions of each [^11^C]HED formulation. Total tracer retention is expressed as percentage of injected dose per gram of tissue, normalized to body weight (% ID/g × BW). To delineate non-specific retention, a subset of animals at 2 and 8 weeks post-STZ or vehicle was injected with the NET inhibitor desipramine (10 mg/kg, ip) 30 min prior to tracer administration. This value was subtracted from the total [^11^C]HED retention to determine specific retention.

### Western immunoblotting

Hearts were removed and flash frozen in liquid nitrogen following decapitation. Hearts were hand powdered under liquid nitrogen and stored at -80°C until use. Total cell lysate was prepared [6,29] and protein content was determined by BCA assay. SDS-PAGE was carried out using 8% polyacrylamide gels with 40 μg of protein loaded for each sample. Protein was transferred to polyvinylidine difluoride membranes. Blocked membranes were incubated in anti-NET (1:750) and anti-GAPDH (1:2000) followed by secondary antibody incubation. Protein was visualized by chemiluminescence Western lighting kit (Perkin Elmer, Waltham, MA, USA) and the Fluorchem HD Imaging System. Analysis was completed with AlphaEase FC software normalizing NET immunoblot densities to GAPDH and expressed as a percentage of controls.

### Tyrosine Hydroxylase Immunostaining

To determine the relative sympathetic nerve density in the myocardium, immunostaining for tyrosine hydroxylase was carried out in paraffin-embedded short axis heart sections as described elsewhere [30-32]. Briefly, after antigen retrieval by citrate, slides were blocked in normal horse serum and incubated for 96 hours at 4°C with 1:100 anti-tyrosine hydroxylase. Slides were then incubated with 1:100 anti-sheep HRP-conjugated secondary antibody and visualized using diaminobenzidine reagent (Vector Labs). Six images from each section were analysed using ImageJ software (National Institutes of Health) to determine the area of positive staining as a percentage of the total field of view.

### Histopathology

Cardiomyocyte health was assessed by histopathology in a subset of rats. Tissues were processed for histological analysis as described elsewhere [4,19]. Hematoxylin and eosin staining assessed cardiomyocyte morphology and fibre size. Masson Trichrome staining was used to determine collagen deposition.

### Statistics

All data are presented as mean ± standard deviation. Differences between STZ-treated hyperglycemic, STZ-treated euglycemic, and vehicle-treated control groups were tested for significance by one-way analysis of variance with Bonferroni's post hoc test using SPSS 17.0 software. Indirect calorimetry data were tested with repeated measures general linear model using SAS 9.0 software. Significance was considered at p < 0.05.

## Results

### Diabetic animal model

**Figure 1:**
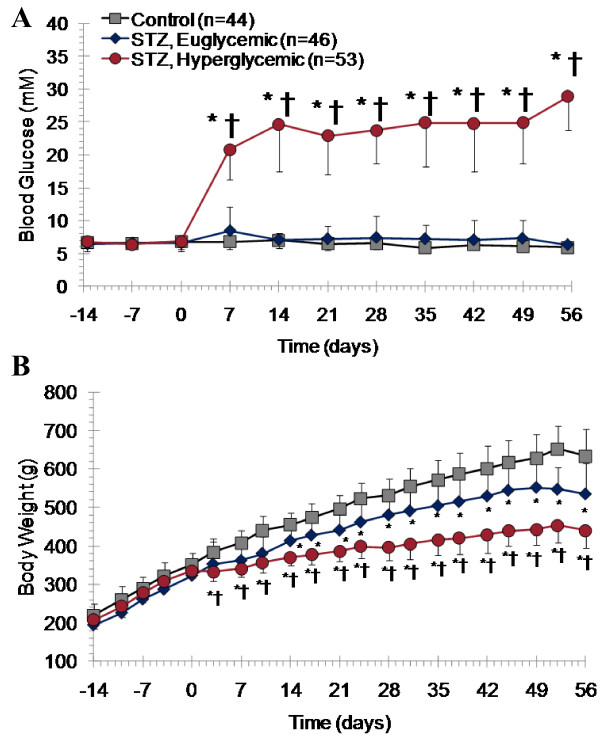
**Blood glucose (**A**) and body weight (**B**) data from high fat diet fed, moderate dose STZ-induced diabetic rats over 8 weeks after STZ or vehicle administration**. Mean ± SD. * p < 0.05 to controls, † p < 0.05 to STZ euglycemic rats, one-way ANOVA, Bonferroni post hoc.

Injection of STZ was successful in inducing overt hyperglycemia in 53.5% of treated rats (n = 53/99), whereas 46 animals maintained normal glucose levels (Figure 1A). After STZ injection, the average fed state blood glucose concentration over 8 weeks in STZ-treated hyperglycemic, STZ-treated euglycemic, and control rats was 20.7 ± 8.7, 7.3 ± 2.7, and 6.5 ± 1.0 mM, respectively. Body weights of all groups were similar until the induction of diabetes, at which point STZ-treated animals displayed stunted weight gain. As diabetes progressed in hyperglycemic rats, further depletion of weight gain was apparent (Figure 1B). One week following treatment and for the remainder of the experiment, STZ-treated hyperglycemic rats tended to consume greater amounts of diet compared to STZ-treated euglycemic and vehicle-treated controls with an average per diem consumption of 568 ± 92, 518 ± 94, and 497 ± 83 kJ, respectively. Heart weight was lower in STZ-treated hyperglycemic rats as compared to controls. A higher heart to body weight ratio was apparent in STZ-treated hyperglycemic rats as compared to both STZ-treated euglycemic and vehicle-treated controls (Table 2).

**Table 2:** Terminal heart and body weights in STZ-treated hyperglycemic, STZ-treated euglycemic, and vehicle-treated control rats

	Control		STZ, Euglycemic		STZ, Hyperglycemic	
	2 Weeks	8 Weeks	2 Weeks	8 Weeks	2 Weeks	8 Weeks
Body Weight (g)	302.4 ± 26.5	584.7 ± 62.2	358.9 ± 22.7*	535.8 ± 43.9*	331.6 ± 30.1	424.6 ± 48.1*^†^
Heart Weight (g)	0.89 ± 0.05	1.46 ± 0.17	1.13 ± 0.14*	1.38 ± 0.11	1.02 ± 0.06*	1.31 ± 0.12*
HW/BW (× 10^-3^)	3.0 ± 0.2	2.5 ± 0.2	3.1 ± 0.3	2.6 ± 0.2	3.1 ± 0.2	3.1 ± 0.3*^†^

Mean ± SD.

* p < 0.05 to age-matched control, † p < 0.05 to age-matched STZ, euglycemic, one-way ANOVA, Bonferroni post hoc

### Blood markers

Hyperglycemic rats displayed progressive hypoinsulinemia, with depletion of insulin levels at 8 weeks but not at 2 weeks post-STZ. Fasted NEFA and triglyceride levels were also significantly lower in STZ-treated hyperglycemic rats as compared to both vehicle-treated and STZ-treated euglycemic controls (Table 3).

**Table 3:** Fasted levels of metabolic blood markers in STZ-treated hyperglycemic, STZ-treated euglycemic, and vehicle-treated control rats

	Control		STZ, Euglycemic		STZ, Hyperglycemic	
Factor	2 Weeks	8 Weeks	2 Weeks	8 Weeks	2 Weeks	8 Weeks
Glucose (mM)	7.0 ± 1.2	6.3 ± 0.4	7.0 ± 1.1	5.9 ± 0.6	24.6 ± 7.2*^†^	28.8 ± 5.1*^†^
Insulin (ng/mL)	0.65 ± 0.41	1.36 ± 0.69	0.67 ± 0.66	0.79 ± 0.57	0.78 ± 0.46	0.33 ± 0.33*^†^
NEFA (nM)	0.20 ± 0.10	0.40 ± 0.13	0.31 ± 0.05	0.20 ± 0.12*	0.30 ± 0.22	0.24 ± 0.14*
Triglyceride (nM)	0.79 ± 0.05	0.81 ± 0.35	0.67 ± 0.17	0.66 ± 0.14	0.80 ± 0.45	0.55 ± 0.29*
NE (ng/mL)	0.10 ± 0.05	0.11 ± 0.06	0.10 ± 0.14	0.10 ± 0.03	0.10 ± 0.06	0.24 ± 0.09*^†^

Mean ± SD.

* p < 0.05 to age-matched control, † p < 0.05 to age-matched STZ, euglycemic, one-way ANOVA, Bonferroni post hoc

### Plasma and cardiac norepinephrine

**Figure 2:**
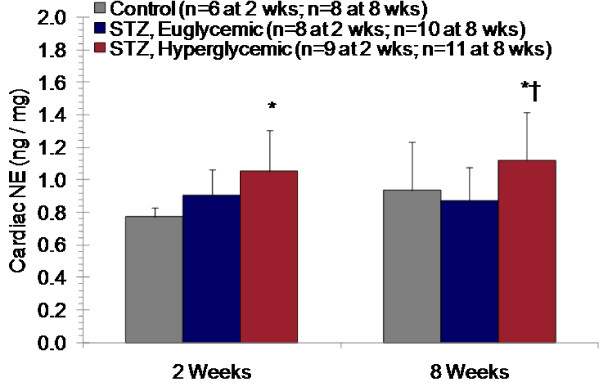
**Cardiac NE content at 2 and 8 weeks after STZ or vehicle administration**. Mean ± SD. * p < 0.05 to controls, † p < 0.05 to euglycemic, one-way ANOVA, Bonferroni post hoc.

Plasma NE concentrations were comparable between the three groups of animals at baseline, but were 2.5 fold higher in STZ-treated hyperglycemic rats at 8 weeks (Table 3). HPLC analysis of cardiac samples revealed a significant 1.4 fold elevation of NE in STZ-treated hyperglycemic rats compared to vehicle-treated controls at 2 weeks post-STZ, and a 1.2 fold elevation at 8 weeks post-STZ. NE levels in STZ-treated euglycemic rats were unchanged compared to vehicle-treated controls at both time points (Figure 2).

### Oral glucose tolerance

**Figure 3:**
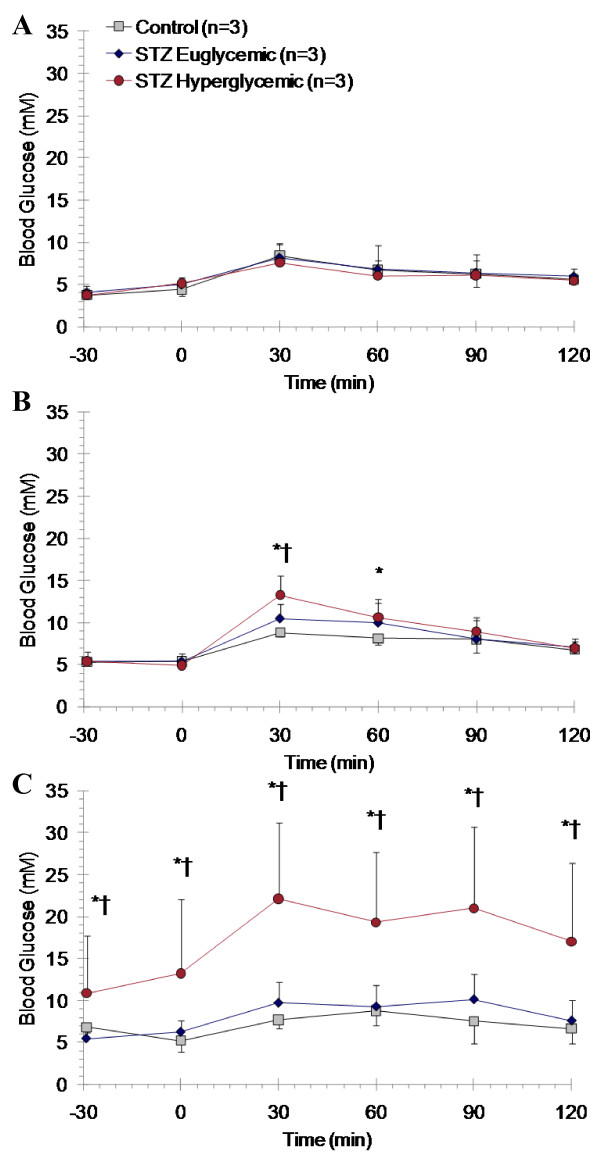
**Oral glucose tolerance testing at baseline (**A**) and at 2 (**B**) and 8 weeks after STZ or vehicle administration (**C**)**. Mean ± SD. * p < 0.05 to controls, † p < 0.05 to STZ euglycemic rats, one-way ANOVA, Bonferroni post hoc.

Prior to induction of diabetes by STZ, there was no difference in clearance of oral glucose load between groups (Figure 3A). However, at 2 and 8 weeks following STZ injection, animals with overt hyperglycemia displayed impaired fasting glucose levels, higher peak glucose after gavage, and a longer time to return to baseline (Figure 3B-C). STZ-treated euglycemic rats showed similar glucose tolerance to vehicle-treated controls. The response of insulin secretion to oral glucose load was ablated in STZ-treated hyperglycemic rats and diminished in STZ-treated euglycemic rats as compared to controls with peak plasma insulin concentrations of 0.4 ± 0.1, 1.4 ± 1.0, and 3.8 ± 1.4 ng/mL at 8 weeks, respectively.

### Euglycemic clamp

Rats at 8 weeks post-STZ were successfully clamped at 5 mM blood glucose during hyperinsulinemic euglycemic clamp under anaesthesia. The glucose infusion rate required to achieve steady state fasted blood glucose concentration was 4.0 ± 2.0, 2.9 ± 1.8, 3.2 ± 0.4 mg kg^-1 ^min^-1 ^in STZ-treated hyperglycemic, STZ-treated euglycemic, and vehicle treated control rats, respectively. The infusion rates for all high fat-fed animals were significantly lower than for chow-fed controls, which had a glucose infusion rate of 25.0 ± 4.2 mg kg^-1 ^min^-1^.

### Indirect calorimetry

**Figure 4:**
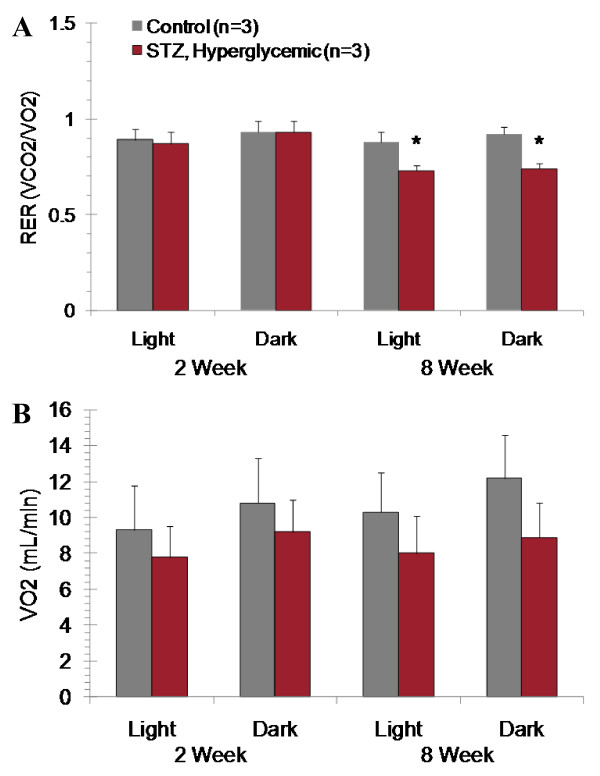
**Indirect calorimetry assessment of oxygen consumption (**A**) and respiratory exchange ratio (**B**) at 2 and 8 weeks after STZ or vehicle administration**. Mean ± SD. * p < 0.05 to controls, repeated measures, general linear model.

STZ-treated hyperglycemic rats oxidized a lower proportion of carbohydrates as compared to vehicle-treated controls after 8 weeks but not 2 weeks of hyperglycemia (Figure 4A). There was a trend toward lower oxygen consumption in STZ-treated hyperglycemic rats as compared to controls (Figure 4B).

### Echocardiography

**Figure 5:**
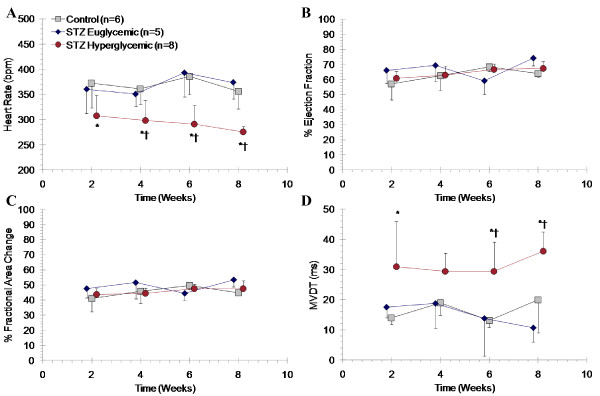
**Serial echocardiographic assessment of left ventricle ejection fraction (**A**), fractional area change (**B**), heart rate (**C**), and mitral valve deceleration time (**D**) at 2, 4, 6, and 8 weeks after STZ or vehicle administration**. Mean ± SD. * p < 0.05 to controls, † p < 0.05 to euglycemic, one-way ANOVA, Bonferroni post hoc.

Measures of systolic and diastolic cardiac function are summarized in Table 4. STZ-treated hyperglycemic rats exhibited significantly lower heart rates than euglycemic and control counterparts (Figure 5A). There was no difference in percent LV ejection fraction or fractional area change between groups (Figure 5B-C). However, STZ-treated hyperglycemic rats demonstrated elevated mean mitral valve deceleration time (Figure 5D) and E/A wave ratio as compared to other groups (Table 4).

**Table 4:** Echocardiography parameters in STZ-treated hyperglycemic, STZ-treated euglycemic, and vehicle treated control rats at 2, 4, 6, and 8 weeks after STZ

	Control				STZ, Euglycemic				STZ, Hyperglycemic			
	2 Weeks	4 Weeks	6 Weeks	8 Weeks	2 Weeks	4 Weeks	6 Weeks	8 Weeks	2 Weeks	4 Weeks	6 Weeks	8 Weeks
EV (d) (μL)	477 ± 135	477 ± 65	431 ± 90	353 ± 43	570 ± 47	457 ± 100	400 ± 67	363 ± 35	435 ± 103	497 ± 117	483 ± 80	369 ± 111
EV (s) (μL)	199 ± 49	184 ± 72	137 ± 35	128 ± 19	192 ± 33	134 ± 39	160 ± 9	95 ± 23	189 ± 57	182 ± 42	162 ± 36	119 ± 37
SV (μL)	278 ± 122	293 ± 30	293 ± 58	225 ± 27	379 ± 81	323 ± 118	240 ± 76	268 ± 21	262 ± 57	314 ± 86	321 ± 52	250 ± 79
CO (mL/min)	105 ± 52	106 ± 15	112 ± 16	81 ± 11	138 ± 47	113 ± 37	93 ± 18	100 ± 13	82 ± 23	91 ± 18	94 ± 19	69 ± 22
IVCT (ms)	62 ± 5	79 ± 13	70 ± 6	64 ± 9	84 ± 5	74 ± 7	65 ± 7	73 ± 6	83 ± 14	81 ± 14	83 ± 15	78 ± 6
IVRT (ms)	22 ± 4	33 ± 5	25 ± 8	32 ± 7	23 ± 0	30 ± 5	31 ± 2	24 ± 6	27 ± 6	25 ± 4	34 ± 15	33 ± 5
A (cm/s)	62 ± 22	77 ± 13	85 ± 33	77 ± 25	59 ± 6	83 ± 22	65 ± 17	97 ± 23	50 ± 13	65 ± 21*^†^	58 ± 39	69 ± 19*^†^
E (cm/s)	98 ± 5	118 ± 23	92 ± 26	98 ± 19	105 ± 5	119 ± 22	92 ± 8	125 ± 42	92 ± 18	104 ± 24	98 ± 28	99 ± 21
E/A Ratio	1.7 ± 0.5	1.6 ± 0.3	1.2 ± 0.4	1.3 ± 0.3	1.8 ± 0.1	1.5 ± 0.2	1.4 ± 0.3	1.3 ± 0.2	1.9 ± 0.6	1.7 ± 0.3	2.0 ± 1.0	1.5 ± 0.3

* p < 0.05 to controls, † p < 0.05 to euglycemic, one-way ANOVA, Bonferroni post hoc

(EV = endocardial volume at diastole (d) or systole (s); SV = stroke volume; CO = cardiac output; IVCT = intraventricular contraction time; IVRT = isovolumic relaxation time; A = transmitral atrial (late) filling velocity; E = transmitral early filling velocity)

Calculations:

EV (d) = (4π/3) × (Endo Major (d)/2) × ((Endo Area (d))/π (Endo Major (d)/2))^2^

EV (s) = (4π/3) × (Endo Major (s)/2) × ((Endo Area (s))/π (Endo Major (s)/2))^2^

SV = EV(d) - EV (s)

EF = SV/EV (d) × 100%

FAC = (Endo Area (d) - Endo Area (s))/Endo Area (d) × 100%

CO = SV × Heart Rate

### [^11^C]HED ex vivo biodistribution

**Figure 6:**
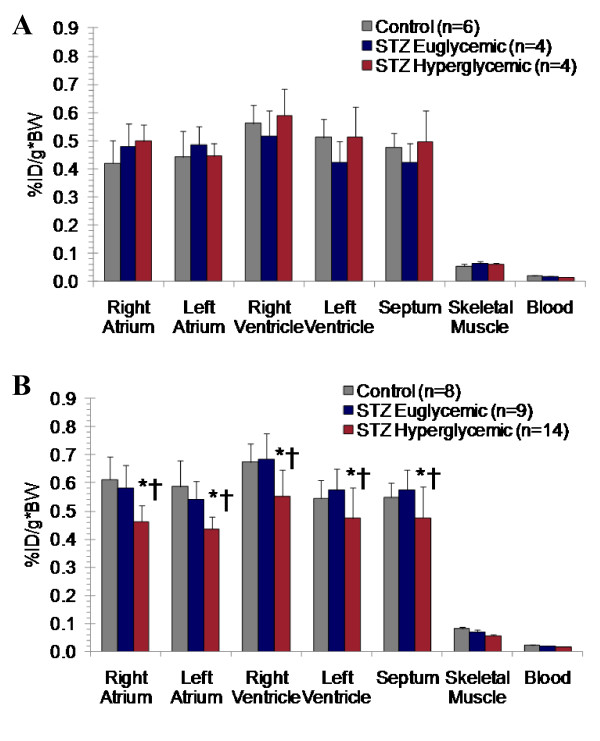
***Ex vivo *biodistribution of [^11^C]HED 30 min post-tracer injection in cardiac regions and skeletal muscle at 2 (**A**) and 8 weeks (**B**) after STZ or vehicle administration**. Mean ± SD. * p < 0.05 to controls, † p < 0.05 to euglycemic, one-way ANOVA, Bonferroni post hoc.

Retention of [^11^C]HED was uniform in myocardium and effectively blocked (81-86%) by pretreatment with desipramine in all groups of animals. At 2 weeks post-STZ, no difference in total or specific myocardial [^11^C]HED accumulation was observed between the groups (Figure 6A). Conversely, 8 weeks following induction of diabetes, hyperglycemic rats exhibited a significant decrease in [^11^C]HED retention as compared to STZ-treated euglycemic and controls (Figure 6B). There was no change in non-specific retention as defined by pretreatment with desipramine. Percent change in total and specific cardiac tracer retention were similar at 13-26% and 12-26%, respectively (Table 5).

**Table 5:** Percent change in total and specific cardiac HED retention compared to vehicle treated controls

Group	Right Atrium	Left Atrium	Right Ventricle	Left Ventricle	Septum
Total Retention					
STZ, Euglycemic	-5 ± 1	-8 ± 1	+2 ± 0	+5 ± 1	+5 ± 1
STZ, Hyperglycemic	-24 ± 4	-26 ± 4	-18 ± 3	-13 ± 3	-13 ± 3
Specific Retention*					
STZ, Euglycemic	-6 ± 1	-12 ± 1	+1 ± 0	+5 ± 1	+5 ± 1
STZ, Hyperglycemic	-24 ± 4	-26 ± 4	-19 ± 3	-12 ± 3	-13 ± 3

Mean ± SD.

*Specific Retention (SR) = Total Retention (TR) - Non-Specific Retention (NSR) [desipramine]

% Change = TR_STZ _- TR_control _/TR_control_

### Western immunoblotting

**Figure 7:**
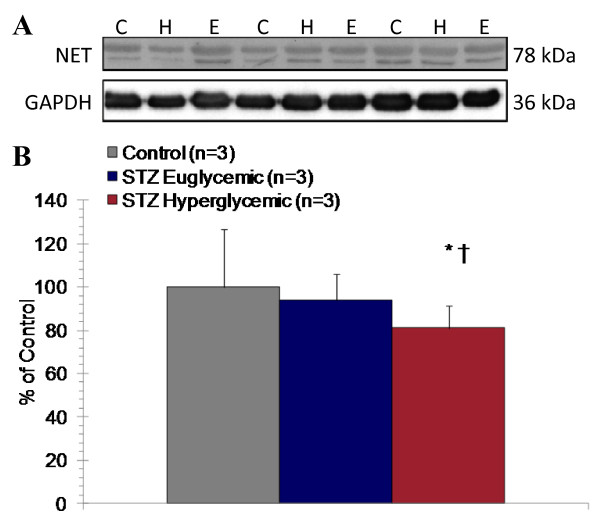
**Representative immunoblots for NET and GAPDH (**A**)**. Relative quantification of immunoblot density between groups 8 weeks after STZ or vehicle administration (**B**). Mean ± SD. * p < 0.05 to controls, † p < 0.05 to euglycemic, two-tailed t-test.

Immunoblots for NET resulted in consistent protein bands at 76 and 78 kDa, as per the manufacturer's documentation (Figure 7A). Relative NET expression normalized to GAPDH was significantly reduced in cardiac lysate of STZ-treated hyperglycemic rats by 17% compared to controls and by 15% to STZ-treated euglycemic rats (Figure 7B).

### Tyrosine Hydroxylase Immunostaining

**Figure 8:**
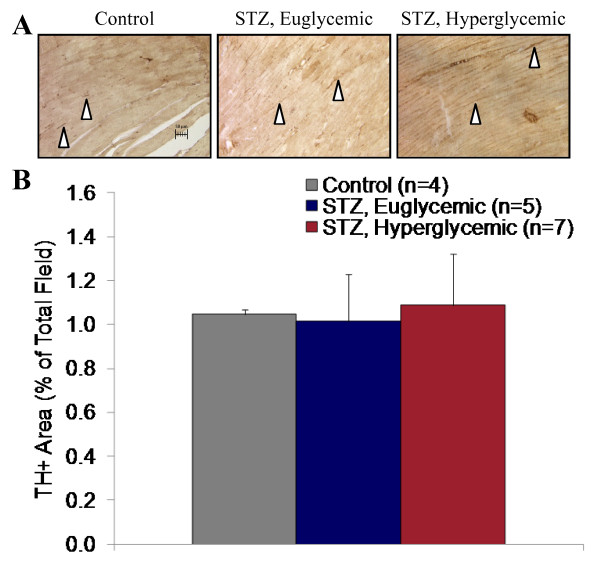
**Sympathetic nerve density assessment by tyrosine hydroxylase immunostaining of left ventricle sections at 8 weeks after STZ or vehicle administration**. Representative slides from vehicle-treated control, STZ-treated euglycemic, and STZ-treated hyperglycemic rats (**A**). 20 × magnification; white arrowheads indicate tyrosine-hydroxylase positive nerve terminals. Relative sympathetic nerve density as percentage tyrosine-hydroxylase-positive area per field of view (**B**).

Tyrosine-hydroxylase-positive nerve endings were identified within the left ventricle in each group (Figure 8A). Quantitative analysis revealed no difference in staining between STZ-treated hyperglycemic, STZ-treated euglycemic, and vehicle-treated controls, suggesting maintained sympathetic innervation (Figure 8B).

### Histopathology

**Figure 9:**
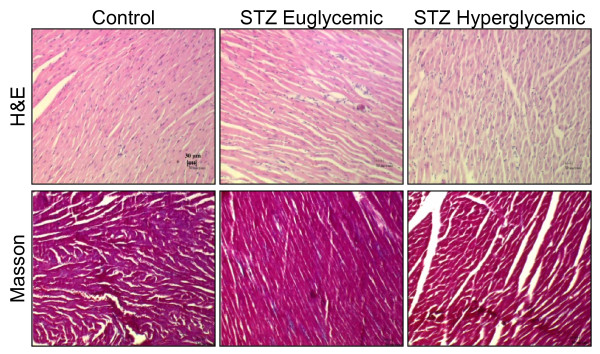
**Histopathology of left ventricle at 8 weeks after STZ or vehicle administration**. Hematoxylin and eosin (H&E) and Masson Trichrome staining in control, STZ euglycemic, and STZ hyperglycemic rats. 20 × magnification.

There were no differences in cardiomyocyte morphology between STZ-treated hyperglycemic, STZ-treated euglycemic, and control rats (Figure 9). Masson Trichrome staining revealed no change in collagen content between groups (Figure 9).

## Discussion

In the present article, we describe a progressive deterioration of cardiac sympathetic nervous integrity in the presence of diastolic left ventricular abnormalities in a rat model of insulin resistance with uncontrolled hyperglycemia. A reduction of 12-26% in myocardial [^11^C]HED retention is paralleled by a modest decrease in relative cardiac NET expression and an increase in plasma and cardiac NE content at 8 weeks of diabetes with no change in sympathetic nerve density. While diastolic function appears to be abnormal, left ventricular ejection fraction is maintained, and histology is unchanged, indicating preserved systolic function.

### Diabetic Animal Model

The animal model used in these experiments mimics certain aspects of clinical type 2 diabetes, exhibiting insulin resistance, sustained hyperglycemia, and progressive hypoinsulinemia [24-26]. The STZ-treated hyperglycemic rats show reduced insulin, NEFA and triglyceride levels as compared to both sets of controls at 8 weeks, inconsistent with some previous reports describing elevated lipid levels [25]. This may reflect the longer duration of insulin resistance and diabetes examined in the present study, 2 to 8 weeks post-STZ as compared to 3-7 days. Glucose tolerance deteriorates over time following STZ injection, as evidenced by greater impairment of fasting glucose and higher peak glucose levels attained in hyperglycemic rats following oral glucose loading. Zhang and colleagues demonstrated a similar result with intravenous glucose tolerance testing in this animal model [24]. High fat feeding has been shown to induce insulin resistance in rodents, a result that is reflected by the current data. Insulin resistance and glucose intolerance is further supported by the indirect calorimetry data, which demonstrate fatty acid as the preferred energy substrate in STZ-treated hyperglycemic rats, as in other diabetic animal models and in patients [33,34]. These diabetic rats lack the obesity and prolonged hyperinsulinemia commonly associated with type 2 diabetes, but permit the evaluation of elevated blood glucose in the presence of high fat diet-induced insulin resistance. Moreover, the inclusion of STZ-treated euglycemic rats, which show a modest development of insulin insufficiency at 8 weeks, suggests that hyperglycemia is the predominant influencing factor in sympathetic dysregulation for this model.

### Cardiac Dysfunction in Diabetic Rats

Development of cardiac dysfunction has been documented in several animal models of type 1 and type 2 diabetes, with decline in systolic and diastolic function, as well as in cardiac output [2,3,18,21,35]. In the present experiments, hyperglycemic rats exhibit some indicators of impaired diastolic function, as measured by consistently higher E/A ratio and prolonged mitral valve deceleration time. Diastolic dysfunction is a common early manifestation of heart disease in diabetic animal models [35] and patients [36]. This is comparable to previous reports in the present animal model and in the Zucker Diabetic Fatty rat model of type 2 diabetes, which exhibit reduced heart rate, prolonged isovolumic relaxation time and increased chamber stiffness [3,18,19,36]. Because diastolic parameters are influenced by the time spent in diastole, the reduction of heart rate observed in STZ-treated hyperglycemic rats poses a complication in interpreting these results. A recent study of STZ-induced diabetes in Wistar rats established that autonomic impairment, as measured by reduced baroreflex sensitivity to phenylephrine, was associated with diastolic dysfunction, characterized both by echocardiographic and left ventricular hemodynamics [21]. Still, further investigation of diastolic function in the present case is warranted.

### Sympathetic Nervous Integrity

In the current study, there is a conclusive reduction in presynaptic sympathetic nervous integrity, consistent with previous reports in animal models of diabetes. Reduced tracer accumulation is present despite an increase in heart to body weight ratio compared to STZ-treated euglycemic and vehicle-treated controls, which in the absence of a physiological alteration to sympathetic nervous integrity would be instead expected to increase. This result suggests that reduced [^11^C]HED retention reflects abnormal sympathetic innervation rather than an artifact of tissue weight. Schmid and colleagues have shown a progressive regional reduction of [^11^C]HED retention in distal left ventricle in intravenous high dose STZ-induced type 1 diabetic rats as compared to non-diabetic controls, associated with elevated NE at 6 months of diabetes and depletion of nerve growth factor (NGF) at 9 months [13]. These data suggest that diabetic autonomic neuropathy associated with reduced cardiac NE content is not present at 8 weeks of diabetes, rather displaying dysregulated sympathetic signaling characterized by elevated NE release. Recently, small animal single photon emission computed tomography (SPECT) imaging using the [^11^C]HED analogue [^123^I]*meta*-iodobenzylguanidine (MIBG) in db/db type 2 diabetic mice demonstrated maintained tracer uptake with enhanced washout rate [8]. These findings are consistent with elevated sympathetic activity without a pronounced decrease in NET density; that is, enhanced local release of catecholamines and vesicle-packaged MIBG. Furthermore, Kiyono and colleagues have suggested that differential factors influence MIBG retention in rat models of type 1 and type 2 diabetes [37,38]. Specifically, in intravenous STZ-induced type 1 diabetic rats, MIBG retention was moderately reduced compared to controls, paralleled by a twofold increase in cardiac NE concentration and no change in NET [38]. By contrast, in Goto Kakizaki non-obese type 2 diabetic rats, the global and regional decrease in MIBG uptake was correlated to no change in cardiac or plasma NE but a 30-45% reduction in NET B_max _as assessed by [^3^H]desipramine binding assay [37]. In the present study, after 8 weeks of uncontrolled hyperglycemia, a modest decrease in apparent NET expression is paralleled by a significant 20% elevation of cardiac NE, 250% elevation of plasma NE, and a significant 12-25% reduction in [^11^C]HED retention. Importantly, the consistent tyrosine hydroxylase immunostaining between groups indicates that sympathetic denervation is not responsible for reduced [^11^C]HED retention. Persistently elevated catecholamines evoke downregulation of cardiomyocyte β-adrenoceptor expression in parallel with NET [39]. We and others have demonstrated that adrenoceptors in diabetic myocardium are reduced by 30-40% [40] and can be restored by insulin treatment [6]. This has important functional consequences, manifesting as impaired calcium transport by sarcoplasmic/endoplasmic reticulum calcium ATPase (SERCA) and loss of excitation-contraction coupling [21,41].

### Sympathetic Nervous System and Heart Disease

Cardiac deterioration is a common endpoint for most diabetic animal models and the most common cause of death in diabetic patients [1]. Studies in isolated perfused hearts of STZ-induced diabetic rats have shown reduced cardiac output, depressed cardiac β-adrenoceptor expression, and partial restoration by treatment with the β-blocker metoprolol [2]. Indeed, Mongillo and colleagues propose that presynaptic sympathetic integrity is proportional to glucose uptake in heart failure [42], suggesting a complementary value in [^11^C]HED retention to myocardial viability. Considered with the present experiments, these data imply a role for sympathetic dysregulation in the progression of cardiac disease in diabetic rats. Continuous β-adrenergic stimulation by infusion of isoproterenol generates cardiac hypertrophy, bradycardia, and slowed end-diastolic relaxation rates [43]. NET knockout mice display elevated cardiac NE, hypertension and tachycardia [44]. Conversely, overexpression of NET has been shown to ameliorate downregulation of β-adrenoceptors and SERCA, and restore ventricle diameters and systolic function in experimental heart failure [45]. Catecholamines have been implicated in the development of cardiovascular dysfunction, acting to dysregulate calcium transport, alter angiotensin II signaling [4,46], increase reactive oxygen species [47], and exert direct toxicity by conversion to quinones [48]. Recent work has suggested that abnormal heart rate and heart rate variability in diabetic rats can be normalized by renal denervation using phenol, though conclusions are preliminary [20]. Cardiac autonomic neuropathy is a common complication of diabetes, associated with depletion of cardiac NE stores and reduced neurotrophin expression. It is conceivable that a period of sympathetic hyperactivity precedes denervation and may provide opportunity for neuronal rescue by NGF therapy, as suggested elsewhere [32].

### Potential of PET in Diabetic Heart Disease

The current experiments suggest that non-invasive PET measures of cardiac sympathetic nervous integrity may have important prognostic value for cardiac events in diabetes. In the ADMIRE HF trial, Jacobson and colleagues demonstrated that patients with the lowest heart to mediastinal ratio in MIBG scintigraphy had ten times higher cardiac mortality than those with preserved neuronal uptake [49]. Similarly, the Detection of Ischemia in Asymptomatic Diabetics (DIAD) trial attempted to identify patients at risk of developing cardiac dysfunction by analyzing myocardial perfusion in diabetic patients with preserved ejection fraction [50]. Patients who showed impaired perfusion were more likely to incur a cardiac event. The present results suggest that analysis of sympathetic nervous integrity may provide a more sensitive marker of early cardiac dysfunction in diabetes. A 3 year follow-up [^11^C]HED imaging study in type 1 diabetic patients demonstrated recovery of [^11^C]HED retention in patients with good glycemic control, and greater defect size with reduced tracer uptake in patients with poor glycemic control [7]. These studies indicate the potential for molecular imaging to stratify diabetic patients for cardiac risk.

### Limitations

A limitation in the use of [^11^C]HED biodistribution to define sympathetic nervous system defects is the inability to delineate the mechanistic cause of the abnormality. Sympathetic nervous dysfunction can manifest both at the nerve terminals, which can be assessed by [^11^C]HED PET, or in the central nervous system with efferent nerve traffic, which cannot. Previous work has demonstrated attenuation of sympathoadrenal responses, originating from higher brain centers [51]. Moreover, direct measurement of neurotrophins was not undertaken in this study, though maintained sympathetic nerve density and elevated cardiac and plasma NE suggest functional sympathetic neurons. While the present work confirms abnormal cardiac sympathetic nervous signaling in sustained hyperglycemia, further research is warranted to more closely define the mechanism of this defect and its relation to diastolic dysfunction.

## Conclusions

Sustained hyperglycemia with insulin resistance in the high fat fed moderate dose STZ rat is associated with cardiac sympathetic dysregulation manifested by elevated plasma and cardiac NE, reduced NET expression, and depleted [^11^C]HED retention with maintained sympathetic nerve density. These findings were observed in conjunction with echocardiographic indicators of diastolic dysfunction and in the absence of systolic dysfunction of the diabetic myocardium. Taken together, these results suggest that non-invasive [^11^C]HED PET may be a useful tool to monitor cardiac sympathetic nervous dysfunction and guide glycemic therapy in diabetic patients.

## Competing interests

The authors declare that they have no competing interests.

## Authors' contributions

JTT performed experiments, contributed to discussion, and drafted the manuscript. JR, MEH, EJS and KJA contributed to discussion and reviewed/edited the manuscript. RSB and JND supervised research, contributed to discussion and writing, and reviewed/edited the manuscript. All authors have read and approved the final manuscript.
